# Identification of stress-responsive transcription factors with protein-bound *Escherichia coli* genomic DNA libraries

**DOI:** 10.1186/s13568-020-01133-0

**Published:** 2020-11-02

**Authors:** Xianqiang Li, Xin Jiang, Meiying Xu, Yun Fang, Yan Wang, Guoping Sun, Jun Guo

**Affiliations:** 1 Guangdong Provincial Key Laboratory of Microbial Culture Collection and Application, State Key Laboratory of Applied Microbiology Southern China, Guangdong Institute of Microbiology, Guangdong Academy of Sciences, Guangzhou, China; 2 Science and Technology Library of Guangdong Province and Guangdong Institute of Science and Technology Information and Development Strategy, Guangzhou, China

**Keywords:** Genomic DNA libraries, Luciferase assay, Transcription factors, ArsR, LexA

## Abstract

Bacteria promoters along with operators are crucial elements in the control of gene expression in microbes in response to environmental stress changes. A genome-wide promoter DNA regulatory library is in demand to be developed for a microbe reporter method to monitor the existence of any given environmental stress substance. In this study, we utilized *Escherichia coli* (*E. coli*) as a model system for the preparation of both cell lysates and genomic DNA fragments. Through enriching protein-bound DNA fragments to construct luciferase reporter libraries, we found that, of 280 clones collected and sequenced, 131 clones contained either the promoter-35 and -10 conservative sequences and/or an operator transcription factor binding sites (TFBS) region. To demonstrate the functionality of the identified clones, five of 131 clones containing LexA binding sequence have been demonstrated to be induced in response to mitomycin C treatment. To evaluate our libraries as a functional screening library, 80 randomly picked up clones were cultured and treated with and without MMC, where two clones were shown to have greater than twofold induction. In addition, two arsenite-responsive clones were identified from 90 clones, one having the well-known ArsR and another having the osmotically inducible lipoprotein (OsmE1). The newly discovered *osmE1* has been quantitatively validated to be induced by arsenite treatment with real-time PCR in a dose response and time course manner. This enriching protein-bound DNA luciferase reporter libraries and functional screening facilitate the identification of stress-responsive transcriptional factors in microbes. We developed functional libraries containing *E. coli* genomic-wide protein-bound DNA as enhancers/operators to regulate downstream luciferase in response to stress.

## Introduction

Microbes are highly adaptable to environmental toxic stress such as heavy metals, pesticides, and polychlorinated biphenyls (PCBs) (Chowdhury et al. 2018; Caine 2012). The adaptation to changes in their environment is controlled by the induction or repression of gene expression (Balleza et al. 2009; Cases et al. 2003). Association or dissociation of a transcription factor (TF) to its DNA binding site is a critical step in the initiation of the transcription of its target gene (Fernandez-López et al. 2015; Rogers et al. 2015). It is vital to identify and characterize genes involved in the response to an environmental stress from the entire genome. This facilitates both the understanding of the mechanisms of gene regulation as well as the identification of the key regulatory elements during environmental adaptation in the host.

Environmental genomic toxic stresses such as certain types of chemical reagents and UV irradiation can cause changes in gene expression and cellular metabolism of microbe (Foster 2007). The distinguishing feature of these genes is the presence within the promoter region of a binding sequence for transcriptional repressors, such as LexA (Butala et al. 2009) and ArsR (Chen et al. 2017). LexA repressor normally is bound to its binding sites, repressing transcriptional expression. In response to any DNA damage, the LexA repressor undergoes dissociation from its binding sequences and activate DNA repair genes (Butala et al. 2009). ArsR is a regulatory protein that controls the expression of the genes involved in arsenical resistance via interaction with the arsenic-responsive operon (Wu and Rosen 1993). Upon arsenic binding, the protein dissociates from the promoter, subsequently activating relevant gene expression (Shi et al. 1994). Nevertheless, many toxic substances and their corresponding genes are not well characterized due to lack of simpler and more efficient methods.

Traditionally, transcription factor binding sites (TFBSs), are identified through approaches such as DNase I footprinting (Brenowitz et al. 1989) and electromobility shift assays (Hellman and Fried 2007), which are limited to the interactions between TFs and single targets. Recently, multiple TFs have been experimentally investigated using the systematic evolution of ligands by exponential enrichment (SELEX) (Ishihama et al. 2016) and chromatin immunoprecipitation with microarray (ChIP-chip) or by sequencing (ChIP-seq) (Galagan et al. 2013). Both ChIP-seq and genomic SELEX require the knowledge of stress-corresponding TFs prior to analysis, with time-consuming and tedious procedures. Recently many microbial genomes have been completely sequenced due to advances in the high-throughput genome sequencing, leading to computational methods to identify transcription factor binding sites (TFBSs) in these microbial genomes, However, computational method cannot identify the location and function of promoter region of a transcription factor (Inukai et al. 2017).

Identification of a specific target’s responsive TFBS is very helpful for the development of bacteria biosensors in detecting a chemical substance and its toxicity. However, most of the current bacteria biosensors utilize the existing substrate-induced promoter and operator regions, such as arsenite detection biosensor with GFP (Zaslaver et al. 2006) and luciferase (Chen et al. 2019) as reporters. For a new and potential toxin without knowing its associated TFs, no global reporter method has been developed yet to identify and determine the associated TFs or TFBSs that are required in the regulation of gene expression.

In this study, we present an innovative high-throughput approach to screen and discover TFBSs in response to a stress substance directly without any prior genome information. Functional libraries have been constructed with enriched protein-bound genomic DNA fragments as enhancer and operators extracted from *E. coli* DH5α, along with downstream luciferase reporter to facilitate functional screening. 74% of the sequenced clones were predicted to contain regulatory TFBS with BPROM program from Softberry (Solovyev and Salamov 2011). From 80 randomly screened clones upon mitomycin C (MMC) treatment, two clones were found to be induced and confirmed to contain LexA binding sites. Furthermore, when screening another 90 clones with arsenite treatment, two clones were shown to be induced and have ArsR binding site, corresponding to *arsR* and *osmE1*. In the paper we newly discovered *osmE1* gene, containing an *arsR* binding motif. The gene expression of *osmE1* was further validated by real-time RT-PCR in a dose–response and time course of arsenite-mediated induction.

## Materials and methods

### Preparation of cell lysate proteins

One mL of *E. coli* DH5α culture was centrifuged at 10,000*g* for 1 min and the pellet was resuspended in 300 μL of lysis buffer (10 mM Tris–HCl, pH 8.0, 0.1 M NaCl, 1 mM ethylenediamine tetraacetic acid (EDTA), and 0.1% (w/v) polyethylene glycol octylphenyl ether (Triton X-100)). 7.5 μL of a freshly prepared lysozyme solution (10 mg/mL in 10 mM Tris–HCl, pH 8.0, final concentration = 0.25 mg/mL) was added and mixed by tapping the tube gently, and the lysis mixture was incubated for 10–20 min at room temperature. After centrifugation, the supernatant was used for filter-binding selection.

### Preparation of Genomic DNA fragments

DH5α cells were collected through centrifugation, resuspended in 200 μL lysis buffer (10 mM Tris–HCl, pH 8.0, 1 mM EDTA, 0.5% SDS) and treated with 20 μg/mL proteinase K for 2 h at 55 °C. Genomic DNA was extracted with phenol and chloroform. The genomic DNA was digested with *Mnl*I, 5′…CCTC(N)7…3′, which recognizes four base pairs and generates one nucleotide protruding end at the 3′ terminus, for 1 h at 37 °C. The genomic DNA fragments were subsequently purified with MinElute Reaction Cleanup Kit (QIAGEN, Hilden, Germany).

### Filter-binding selection of protein-bound DNA fragments

Five μL cell lysate (2–10 μg) was mixed with 15 μL 2X Binding buffer (40 mM 4-(2-hydroxyethyl)-1-piperazineethanesulfonic acid (HEPES), pH 7.6, 20 mM ammonia sulfate, 2 mM dithiothreitol (DTT), 20 mM KCl, and 0.4% Tween-20), *Mnl*I-digested 5 μL genomic DNA and 5 μL ddH_2_O in a PCR tube. After incubation at room temperature for 30 min, we loaded 30 μL binding mixture onto a prewashed filter assay column and incubated on ice for 20 min. The column is a nitrocellulose-based filter system, which can bind proteins and protein-DNA complex. After four times washing with Filter washing buffer to remove free DNA oligos, the bound DNA fragments were eluted with elution buffer (0.5% SDS). The eluted DNA fragments were subsequently used for generating libraries.

### Construction of genomic libraries

**Fig. 1:**
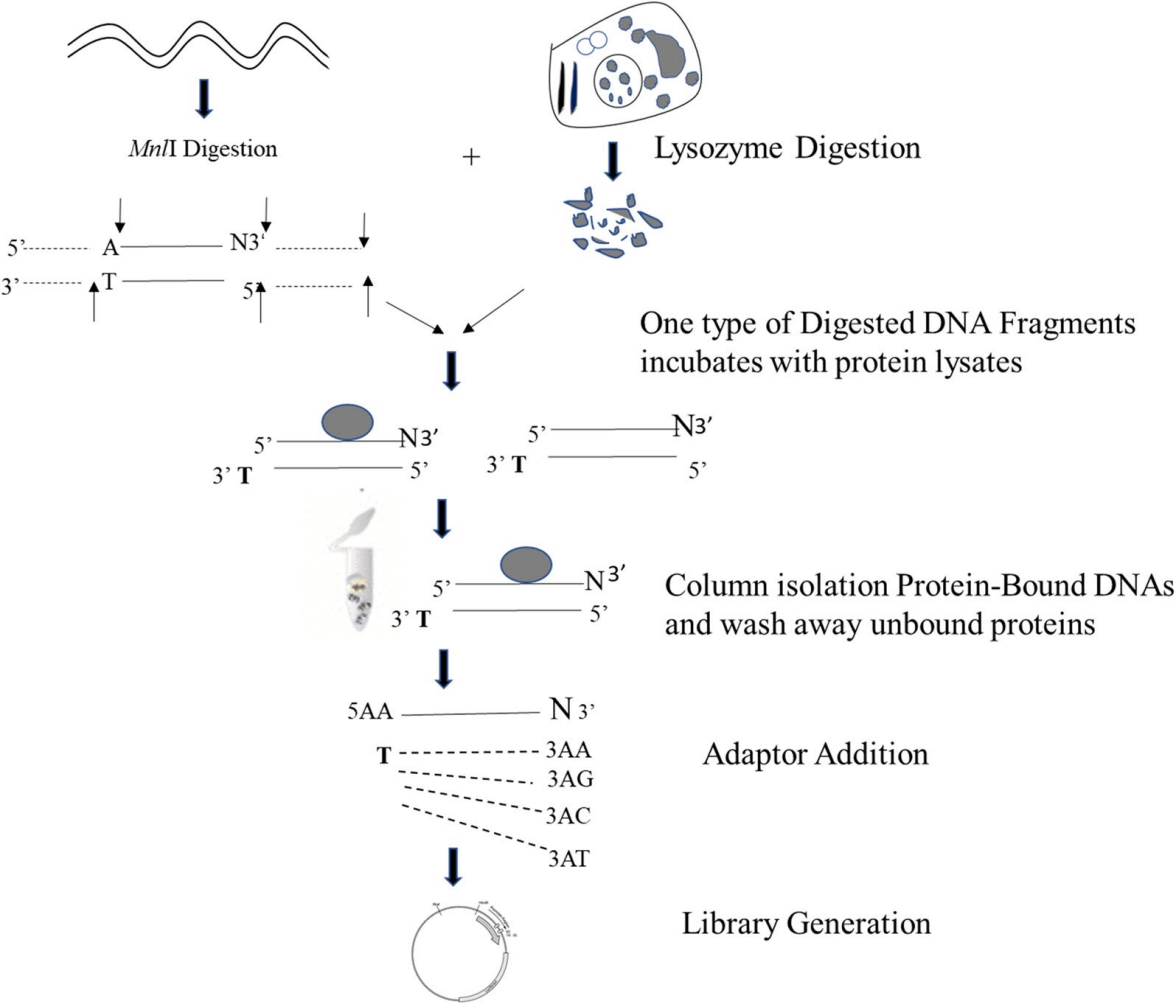
**a** Schematic diagram of separation of protein-binding genomic DNA fragments for library construction. Genomic DNA was prepared from DH5α with proteinase K digestion followed by phenol and chloroform extraction. It was then sheared with *Mnl*I digestion. Proteins were also extracted from DH5α cells and incubated with the genomic DNA fragments to allow formation of protein/DNA complexes, which were able to retain on a filter column and to separate from the unbound DNA by the following washing steps. The protein-bound DNA fragments were then eluted and used for construction of libraries. **b** 8 different adaptors were made, AA5, AG5, AC5, and AT5, for ligating to 5′ end of *Mnl*I fragments, and AA3, AG3, AC3, and AT3 for ligation to 3′ terminus of *Mnl*I fragments

The eluted protein-bound DNA fragments were ligated with adaptors. *Mnl*I digested fragments may have multiple nucleotide possibilities at the 3uterminus. Two basic sequences for making adaptors were selected to avoid cross hybridization with *E. coli* genome, 5′ATGGATAGGTCGGTGA3′ or 5′GACGCACCTTGAGGC3′. The double strand adaptors were designed and synthesized to match all possible fragments generated by *Mnl*I-digestion (Fig. 1) and two DNA oligos were annealed to form the double strand adaptors with different protruding ends respectively. The oligos were designed and synthesized: (F1T 5′TCACCGACCTATCCAT-T3′, F2T 5′GCCTCAAGGTGCGTC-T3′, F1A 5′TCACCGACCTATCCAT-A3′, F2A 5′GCCTCAAGGTGCGTC-A3′, F1C 5′TCACCGACCTATCCAT-C3′, F2C 5′GCCTCAAGGTGCGTC-C3′, F1G 5′TCACCGACCTATCCAT-G3′, and F2G 5′GCCTCAAGGTGCGTC-G3′). F1 and F2 were annealed with R1S: 5′ATGGATAGGTCGGTGA3 ar R2S 5′GACGCACCTTGAGGC3′ accordingly to form eight adaptors: 5AA, 5AG, 5AC, 5AT, 3AA, 3AG, 3AC, and 3AT (Table 1). After ligation of adaptors with DNA fragments, 16 combinations were amplified by 10 PCR cycles with a forward primer introduced with XbaI sequence and a reverse primer with *Hin*dIII sequence. The amplified products were digested with *Xba*I and *Hin*dIII and cloned into pACYC-Luc vector, which was modified in our previous publication (Chen et al. 2019), originally derived from pACYC184 (New England Biolabs, Ipswich, MA, USA) to generate 16 libraries (AA, AT, AC, AG; TA, TT, TC, TG; CA, CT, CC, CG; GA, GT, GC, GG) listed in Table 1. After transformation, the clones (colonies) were selected on ampicillin plates, and plasmid DNAs from 280 clones were subsequently either prepared and sequenced, or directly conducted induction luciferase screening assay.

**Table 1:** Sixteen genome libraries generated from combination of eight adaptors sequences randomly digested by *Mnl*I restriction enzyme

No	Genome library names	Adaptor combination^a^	5′Adaptor name	5′ Adaptor sequence	3′ Adaptor name	3′ Adaptor sequence
1	AA	5AA-3AA	5AA	5′ ATGGATAGGTCGGTGA 3′ (R1S) 3′**A**-TACCTATCCAGCCACT 5′ (F1A)	3AA	5′ GACGCACCTTGAGGC 3′ (R2S) 3′**A-**CTGCGTGGAACTCCG 5′ (F2A)
2	AT	5AA-3AT			3AT	5′ GACGCACCTTGAGGC 3′ (R2S) 3′**T-**CTGCGTGGAACTCCG 5′ (F2T)
3	AG	5AA-3AG			3AG	5′ GACGCACCTTGAGGC 3′ (R2S) 3′**G-**CTGCGTGGAACTCCG 5′ (F2G)
4	AC	5AA-3AC			3AC	5′ GACGCACCTTGAGGC 3′ (R2S) 3′**C-**CTGCGTGGAACTCCG 5′ (F2C)
5	TA	5TA-3AA	5TA	5′ ATGGATAGGTCGGTGA 3′ (R1S) 3′**T**-TACCTATCCAGCCACT 5′ (F1T)	3AA	5′ GACGCACCTTGAGGC 3′ (R2S) 3′**A-**CTGCGTGGAACTCCG 5′ (F2A)
6	TT	5TA-3AT			3AT	5′ GACGCACCTTGAGGC 3′ (R2S) 3′**T-**CTGCGTGGAACTCCG 5′ (F2T)
7	TG	5TA-3AG			3AG	5′ GACGCACCTTGAGGC 3′ (R2S) 3′**G-**CTGCGTGGAACTCCG 5′ (F2G)
8	TC	5TA-3AC			3AC	5′ GACGCACCTTGAGGC 3′ (R2S) 3′**C-**CTGCGTGGAACTCCG 5′ (F2C)
9	GA	5GA-3AA	5GA	5′ ATGGATAGGTCGGTGA 3′ (R1S) 3′**G**-TACCTATCCAGCCACT 5′ (F1G)	3AA	5′ GACGCACCTTGAGGC 3′ (R2S) 3′**A-**CTGCGTGGAACTCCG 5′ (F2A)
10	GT	5GA-3AT			3AT	5′ GACGCACCTTGAGGC 3′ (R2S) 3′**T-**CTGCGTGGAACTCCG 5′ (F2T)
11	GG	5GA-3AG			3AG	5′ GACGCACCTTGAGGC 3′ (R2S) 3′**G-**CTGCGTGGAACTCCG 5′ (F2G)
12	GC	5GA-3AC			3AC	5′ GACGCACCTTGAGGC 3′ (R2S) 3′**C-**CTGCGTGGAACTCCG 5′ (F2C)
13	CA	5CA-3AA	5CA	5′ ATGGATAGGTCGGTGA 3′ (R1S) 3′**C**-TACCTATCCAGCCACT 5′ (F1C)	3AA	5′ GACGCACCTTGAGGC 3′ (R2S) 3′**A-**CTGCGTGGAACTCCG 5′ (F2A)
14	CT	5CA-3AT			3AT	5′ GACGCACCTTGAGGC 3′ (R2S) 3′**T-**CTGCGTGGAACTCCG 5′ (F2T)
15	CG	5CA-3AG			3AG	5′ GACGCACCTTGAGGC 3′ (R2S) 3′**G-**CTGCGTGGAACTCCG 5′ (F2G)
16	CC	5CA-3AC			3AC	5′ GACGCACCTTGAGGC 3′ (R2S) 3′**C-**CTGCGTGGAACTCCG 5′ (F2C)

^a^ Each adaptor was abbreviated with 5AA (5′ adaptor A), 5AT (5′ adaptor T), 5AG (3′ adaptor G), etc. 5′ adaptors are annealed with shared sequence R1S while 3′ adaptors were annealed with sharded sequence R2S. Each 5′ adaptor was combined with each 3′ adaptor to create a total of 16 genome libraries

### Luciferase assay

For the screening assay, 80-90 individual colonies were picked and inoculated in 600 μL LB media supplied with 25 μg/mL chloramphenicol, and incubated for 12–16 h at 37 °C in the corresponding wells of 96 well deep plate with vigorous shaking. The overnight culture was diluted 1:50 in a new 96 well deep plate with pre-warm and fresh-prepared 600 μL LB media supplied with chloramphenicol. The diluted cells were cultured for an additional 4 h at 37 °C until the optical density (O.D.) reached 0.5. Cells were treated with or without MMC, or sodium arsenite (AsIII) at 37 °C. 20 μL of induced culture was mixed with 50 μL luciferase substrate, and the luciferase activity was measured with Veritas Microplate Luminometer (Tuner Biosystems, Sunnyvale, CA, USA). For an individual clone assay, a plasmid was transformed into DH5α, and a single colony was inoculated in 2 mL LB media 25 μg/mL chloramphenicol for 12–16 h at 37 °C in an individual tube, with the rest of steps being the same as the screening assay and treatment following the description in the results.

### Real-time RT-PCR

A single DH5α colony with the OsmE1 promoter containing clone was cultured overnight and diluted at 1:50 with LB before with MMC treatment in a time course and dose response manner (the detail see in result). Total RNA was prepared with Monarch Total RNA Miniprep Kit (New England Biolabs, Ipswich, MA, USA) with DNAase treatment to remove residual DNA. Integrity of RNA was assessed by electrophoresis. RNA concentration was determined with Qubit™ RNA BR Assay Kit (Thermo Fisher Scientific, Waltham, MA, USA) in Qubit 2.0 Fluorometer according to manufacturer’s instructions. Extracted RNA (400 ng) was reverse transcribed to cDNA with AMV Reverse Transcriptase (Life Science Advanced Technology, St Petersburg, FL, USA). The primers for the target gene OsmE1 and three reference genes were designed with vector NTI (Thermo Fisher Scientific, Waltham, MA, USA) and using Primer-BLAST (NCBI, USA) and synthesized at IDT (Integrated DNA Technologies, Coralville, IA, USA). The primer specificity was confirmed by 2% agarose gel electrophoresis.

SYBR green-based real-time PCR was performed with ABI PRISM 7000 sequence detection system. 20 μL of PCR reaction was prepared based on Q5 DNA polymerase system (New England Biolabs, Ipswich, MA, USA) with 1X SYBR Green, 1X ROX dye (Roche, Basel, Switzerland), 1 μM forward and reverse primer. The amount of cDNA used in each qPCR reaction was: 1 μL for target gene *osm*E1, 1 μL for reference genes, *gry*A and mGOD, and 0.6 μL of 1:100 diluted cDNA for 16S rRNA. These were pre-determined by testing serial dilutions of cDNA samples to achieve the threshold cycle (Ct) values of the three reference genes similar to that of the target gene. We ran the PCR reaction at 50 °C for 2 min and 98 °C for 5 min, followed by 40 cycles at 98 °C for 15 s, 55 °C for 30 s, and 72 °C for 30 s. A dissociation stage was then performed as follows: 95 °C for 15 s, 60 °C for 20 s, and 95 °C for 15 s. All samples were run in duplicate, and the mean Ct values for each trial were calculated. ΔCt was then calculated as the difference between target gene and the geometric mean of three reference genes. ΔΔCt was obtained by normalizing the ΔCt values of the treatments to the ΔCt value of the control without treatment. Finally, relative target gene expression values were calculated with $2^{ - \Delta \Delta Ct}$ (Livak and Schmittgen 2001).

## Results

This screening libraries consist of enriching protein-bound genomic DNA fragments and downstream luciferase reporters. The DNA fragments were generated based on the protein/DNA complex formation and protein/DNA complex separation (Fig. 1). To construct these libraries, *E. coli* genomic DNA was digested with a restriction enzyme *Mnl*I that recognizes non-palindromic nucleotide sequence 5n/DNA comple (Kriukiene et al. 2005), each fragment with one protruding nucleotide at 3cleot with four possibilities: A G, C, and T. If there were DNA fragments containing promoters or TFBS and their corresponding DNA binding proteins in DH5α lysates such as Sigma 70 or TFs, protein/DNA complexes were formed. The enriched protein-bound DNA fragments were obtained and utilized to generate 16 libraries to contain all of promoter and operator regions of genomic DNA. Additionally, these libraries also are functional libraries with luciferase reporter gene. Once the TFs bind on the regulatory DNA regions of libraries, release repressor, and initiate the transcription of luciferase gene. Through measurement of luciferase activities, the clones containing regulatory DNA in response to a treatment.

In order to evaluate libraries with useful TFBS information, approximately 560 clones were obtained from the transformation of these libraries. Of these clones, we selected 280 for sequencing and generated 178 sequences with promoter region sizes around 70–300 bp. First, we analyzed these sequences with a computational analysis of promoter regions and TFBSs. Prokaryotic transcription is performed by RNA polymerase that contains four catalytic subunits and a sigma regulatory subunit. Seven total distinct sigma factors bind a set of promoter sequences and different sigma factors binding sites. The conservative sequences can be found between -10 base pairs and -35 base pairs upstream of the transcription start site in the promoter regions and TFBSs where located upstream of the promoter region acting as an enhancer or a repressor. Using the computer program BPROM (Solovyev and Salamov 2011), we found only 54 sequences having a-10 and -35 bp sigma factor. 71 have at least one TFBS and sigma factors and 6 only contain TFBS without sigma factor. A total of 131 out of 178 clones contain either promoter sequences or TFBSs, or both (shown in Table 2). The sequencing analysis showed that some promoter sequences displayed multiple TFBSs, such as *elbB* containing RpoD18, LexA, GLP, ArcA, FimZ and ArgR, while some had only one TFBS such as *dtpD* containing only LexA. This study revealed a total of 35 unique TFs. Each TF was predicted by BPROM based on its consensus binding element, but the binding sequence on a specific promoter region may be different, which is the reason why we obtained much more the binding sequences than the number of unique TFs (Table 3).

**Table 2:** List of clones containing with -35 and -10 conservative sequences or TF binding sequences

Gene names	Clone ID	Library	Encoded function	Insert length (bp)	TFBS
*aor*	141	TC	Aldehyde ferredoxin oxidoreductase	279	Sigma70
*araH*	28	GA	l-arabinose ABC transporter permease	167	Sigma70, DnaA
*arcB*	47	AC	Sensor histidine kinase	258	Sigma 70, Ihf
*argB*	80	AC	Acetylglutamate kinase	225	Sigma70, ArgR2, Ihf
*arsB*	65, 85, 108, 114	AG, CA CT, TT	Arsenite/antimonite:H(+) antiporter	320	Sigma70
*bglH*	72	AT	Carbohydrate-specific outer membrane porin	244	Sigma70, PurR
*bsl78*	107	CT	DNA (cytosine-5-)-methyltransferase	218	Sigma 70
*cas1e*	18	GG	Type I-E CRISPR-associated protein	186	Sigma70, RpoS17
*casA*	37	GG	CRISPR system Cascade subunit	158	Sigma70, RpoS17
*chbC*	73	AT	*N*,*N*’-diacetylchitobiose-specific PTS enzyme IIC	158	Fur, RpoD17
*chbG*	70, 91	AT	Chitin disaccharide deacetylase	157	Sigma70
*clk_1237*	177	TA	ADP-ribosylglycohydrolase family protein	124	Sigma70
*ctk*	93	CA	DUF4297 domain-containing protein	135	Sigma70, RpoD15, metJ
*ctpA3*	144	TC	Carboxyl-terminal-processing peptidase 3, chloroplastic	158	Sigma70
*cydD*	145	TC	Cysteine/glutathione ABC transporter permease/ATP-binding protein	167	Sigma70
*dgc*	122	TT	Diguanylate cyclases	105	Sigma70, RpoD17
*dtpD*	137	TC	Dipeptide permease	213	Sigma70, LexA
*eamA*	74	AT	Cysteine/*O*-acetylserine exporter EamA	184	Sigma70
*ecm18*	150	TG	Class I SAM-dependent methyltransferase	270	Sigma70, Crp, SoxS
*ef2563*	35	GG	Selenium-dependent molybdenum hydroxylase system protein	133	Sigma70
*egc82*	33	GG	d-hexose-6-phosphate mutarotase	139	Sigma70
*elbB*	138	TC	Isoprenoid biosynthesis glyoxalase	328	Sigma70, Irp,RpoH2, LexA, ArcA, argR, GlpR:
*f0f1*	171	TA	ATP synthase subunit delta	134	Sigma70
*fimZ*	165	TA	Fimbriae biosynthesis transcriptional regulator	96	Sigma70, PurR, LexA1, LexA2, PurR
*frdA*	117	TT	Fumarate reductase flavoprotein subunit	357	Sigma70
*frsA*	1, 81	GA, AC	Esterase	193	Sigma70
*ftr1*	3, 4, 11, 12, 13, 14, 15, 21, 27, 57, 62, 95, 97, 110, 111, 112, 126, 154	GA, GC, GT, AC, AG, CG, CT, TC, TG	Iron permease	145	Sigma70, RpoD16, MetJ
*gaf*	6, 24	GA, TC	GAF domain-containing protein	262	Ihf
*gltT*	86	CA	Cation:dicarboxylase symporter familytransporter	263	Sigma70, RpoD17, OmpR
*gshB*	59	AG	Glutathione synthase	208	Sigma70, Fis
*hcaE*	30	GA	3-Phenylpropionate/cinnamic acid dioxygenase subunit alpha	140	Sigma70, DnaA
*hemK*	147	TC	Peptide chain release factor N(5)-glutamate	366	Sigma70
*hflC*	63	AG	Protease modulator	320	Sigma 70
*hipA*	42	AA	Type II toxin-antitoxin system serine/threonine protein kinase toxin	230	Sigma70, RpoD17
*hjr*	22	GG	Holliday junction resolvase	432	RpoD17
*hsdR*	157	TG	Type I restriction-modification system endonuclease	278	Sigma70
*IcsA*	153	TG	Outer membrane protein IcsA autotransporter precursor	196	Sigma70, RpoD17, SoxS
*IIA*	55	AC	PTS mannitol transporter subunit	154	Sigma70, Ihf. ArgR2, RpoD17
*kch*	53	AC	Voltage-gated potassium channel	160	Sigma70, MetJ, RpoH2
*kdo*	170	TA	3-Deoxy-manno-octulosonate cytidylyltransferase	119	Sigma70, LexA, RpoD18, PurR
*kup*	155	TG	Low affinity potassium transporte	154	Sigma70
*lpfC*	136, 178	TC, TA	Fimbrial biogenesis outer membrane usher protein	155	Sigma70
*maaFP003_1916*	31	GA	Si-specific NAD(P) (+) transhydrogenase	381	Sigma70
*maeB*	94, 116	CA, TT	NADP-dependent oxaloacetate-decarboxylating malate dehydrogenase	121	Sigma, RpoD17, ArgR
*mcrB*	39	GG	5-Methylcytosine-specific restriction enzyme B	128	Sigma70, RpoD17, Irp, Fnr, NagC
*mhpR*	127	TC	DNA-binding transcriptional activator	284	Sigma70, RpoD17, ArgR, ArcA
*mnmC*	69	AT	5-Methylaminomethyl-2-thiouridine biosynthesis bifunctional protein	182	Sigma70
*motA*	9	GC	Flagellar motor stator protein 98	98	FliA, MotAB, CheAW, CpxR
*msyB*	120	TT	Acidic protein	308	Sigma70, SoxS
*mukF*	20	GG	Chromosome partition protein	123	Sigma70, RpoD17, Irp, RpoH2, Fnr, NagC
*narG*	159	TG	Nitrate reductase subunit alpha	154	Sigma70, ArgR
*narI*	10	GC	Respiratory nitrate reductase subunit gamma 195	195	Sigma70, Crp
*nikC*	45	AA	Nickel ABC transporter permease subunit	164	Sigma70, RpoD16
*nrdD*	175	TA	Anaerobic ribonucleoside-triphosphate reductase	350	Sigma70
*nuoE*	158	TG	NADH-quinone oxidoreductase subunit NuoE	222	Sigma70, Crp
*pntA*	8, 50	GA	Si-specific NAD(P) transhydrogenase	415	Sigma70
*rase*	56	AC	4-Hydroxybenzoate octaprenyltransfe	178	Sigma, PurR
*rayT*	88	CA	REP-associated tyrosine transposase	248	Sigma70
*rep*	139	TC	ATP-dependent DNA helicase Rep	185	Sigma70
*rpoS*	156	TG	RNA polymerase sigma factor	315	Sigma70, Crp
*rppH*	169	TA	RNA pyrophosphohydrolase	396	Sigma70, NarP
*rrl*	61	AG	23S ribosomal RNA	153	Crp, RpoD15
*sanA*	67	AT	Outer membrane permeability protein	193	Sigma 70
*sdr*	149	TG	Short-chain dehydrogenase	99	Sigma70
*secA*	167	TA	Preprotein translocase subunit SecA	161	Sigma70, RpoD17, Ihf
*sgr*	132	TC	Helix-turn-helix domain-containing protein	199	Sigma70, ArgR2
*speF*	90	CA	Ornithine decarboxylase SpeF	128	Sigma70
*spy*	105	CT	ATP-independent periplasmic protein-refolding chaperone	233	Ihf, Fis, lrp,
*tesB*	173	TA	Acyl-CoA thioesterase II	133	Sigma70, RpoD16
*thiP*	99	CT	Thiamine/thiamine pyrophosphate ABC transporter permease	210	Sigma70, OmpR
*tolC*	115	TT	Outer membrane channel protein	128	ArgR
*trpS/pgp*	123	TT	Tryptophan–tRNA ligase Phosphoglycolate phosphatase	115	Sigma70
*tyrR*	134	TC	Transcriptional regulator	147	RpoD19, RpoD17, Crp, OmpR, MetR
*ucpA*	100	CT	SDR family oxidoreductase	133	Sigma70, Fnr
*ugdH*	121	TT	UDP-glucose 6-dehydrogenase	294	Sigma70, FlhCD, RpoH2, RpoD17, Fnr, lrp
*uhpC*	16	GG	MFS transporter family glucose-6-phosphate receptor	174	Sigma70, ArgR
*wbbI*	5, 29	GA	Beta-1,6-galactofuranosyltransferase	200	Sigma70
*wcaL*	129	TC	Colanic acid biosynthesis glycosyltransferase	290	Sigma70
*weel*	24, 75	GT	Beta-1,6-galactofuranosyltransferase	217	Sigma70, RpoD17, RpoD16,RpoD17,RpoD17
*wzc*	109	CT	Tyrosine-protein kinase	179	Sigma70
*ybaT*	89	CA	Amino acid permease	171	ArgR2, Ihf, ArcA
*ybjX*	49	AC	DUF535 domain-containing protein YbjX	145	Sigma70
*ycbV*	148	TG	Putative fimbrial-like adhesin protein	110	Sigma70, Crp, RpoD15, PhoB, RpoD17, lrp
*ychE*	131	TC	NAAT family transporter	258	Sigma70, RpoD16, lrp, SoxS, TyrR, GlpR, RpoD18
*ydhW*	23, 25, 40	GT	Oxidoreductase	183	Sigma70
*ydiV*	52	AC	EAL domain-containing protein bacteria	241	Sigma70, Crp
*yeaW*	101	CT	Carnitine monooxygenase subunit	179	Sigma 70
*yedE/fdhT*	48	AC	Selenium metabolism membrane protein	110	Sigma70
*yeeJ*	119	TT	Inverse autotransporter adhesin	182	Sigma70
*yeiH*	135	TC	YeiH family putative sulfate export transporter	114	Sigma70, OxyR, arcA, Fnr, RpoD18, TyrR, Fnr, DeoR, Ihf, ArgR2
*yfeX*	54	AC	Porphyrinogen peroxidase	167	Sigma70
*yhdP*	46	AA	AsmA2 domain-containing protein	200	Sigma70
*yidR*	125	TC	DUF3748 domain-containing galacturonate catabolism protein	174	Sigma70
*yihG*	151	TG	Putative acyltransferase	186	Sigma70
*yneE*	98	CG	Bestrophin family inner membrane protein	120	Sigma70
*ypfG*	64	AG	DUF1176 domain-containing protein	328	Sigma70, RpoH3
	26	GT	Hypothetical protein	211	Sigma 70, ArgR2, Crp
	152	TG	Non-coding, Pseudo genes	186	Sigma70*, Fis, Fnr, LexA*
	161	TG	Unknown	626	Sigma70

**Table 3:** All 35 predicted TFBS with regulation of downstream genes

Number	TFBS	Corresponding genes
1	RpoD16	*ftr1, weel, nikC, narl, ychE, tesB*
2	MetJ	*ftr1, kch, ctk*
3	Ihf	*gaf, arcB, IIA, argB, ybaT, spy, yeiH, secA*
4	FliA	*motA*
5	MotAB-CheAW	*motA*
6	CpxR	*motA, ftr1*
7	Crp	*narl, ydiV, rrl, tyrR, ycbV, ecm18, rpoS, nuoE*
8	ArgR	*uhpC, maeB, tolC, mhpR, elbB, narG,*
9	RpoS17	*cas1e, casA,*
10	RpoD17	*hjr, mukF, weel, mcrB, hipA, llA, chbC, gltT, maeB,* etc.
11	Lrp	*mukF, spy, ugdH, ychE, elbB, ycbV*
12	RpoH2	*mukF, kch, ugdH, elbB,*
13	Fnr	*mukF, mcrB, ucpA, ugdH, yeiH*
14	NagC	*mukF, mcrB*
15	ArgR2	*IIA, argB, ybaT, ftr1, sgr, yeiH*
16	DnaA	*araH, hcaE*
17	Fis	*gshB, spy,*
18	RpoD15	*rrl, ctk, ftr1, ycbV*
19	RpoH3	*ypfG,*
20	PurR	*rase, bglH, fimZ, kdo*
21	SoxS	*narl, msyB, ychE, ecm18, IcsA*
22	OmpR	*gltT, thiP, ftr1, tyrR*
23	ArcA	*mhpR, yeiH, elbB, ybaT,*
24	FlhCD	*ugdH*
25	TyrR	*ychE, yeiH*
26	GlpR	*ychE, elbB*
27	RpoD18	*ychE, yeiH, kdo*
28	MetR	*tyrR*
29	OxyR	*yeiH*
30	DeoR	*yeiH*
31	LexA	*dtpD, elbB, fimZ, kdo, pseuo*
32	PhoB	*ycbV*
33	LexA1	*fimZ*
34	LexA2	*fimZ*
35	NarP	*rpph*

**Fig. 2:**
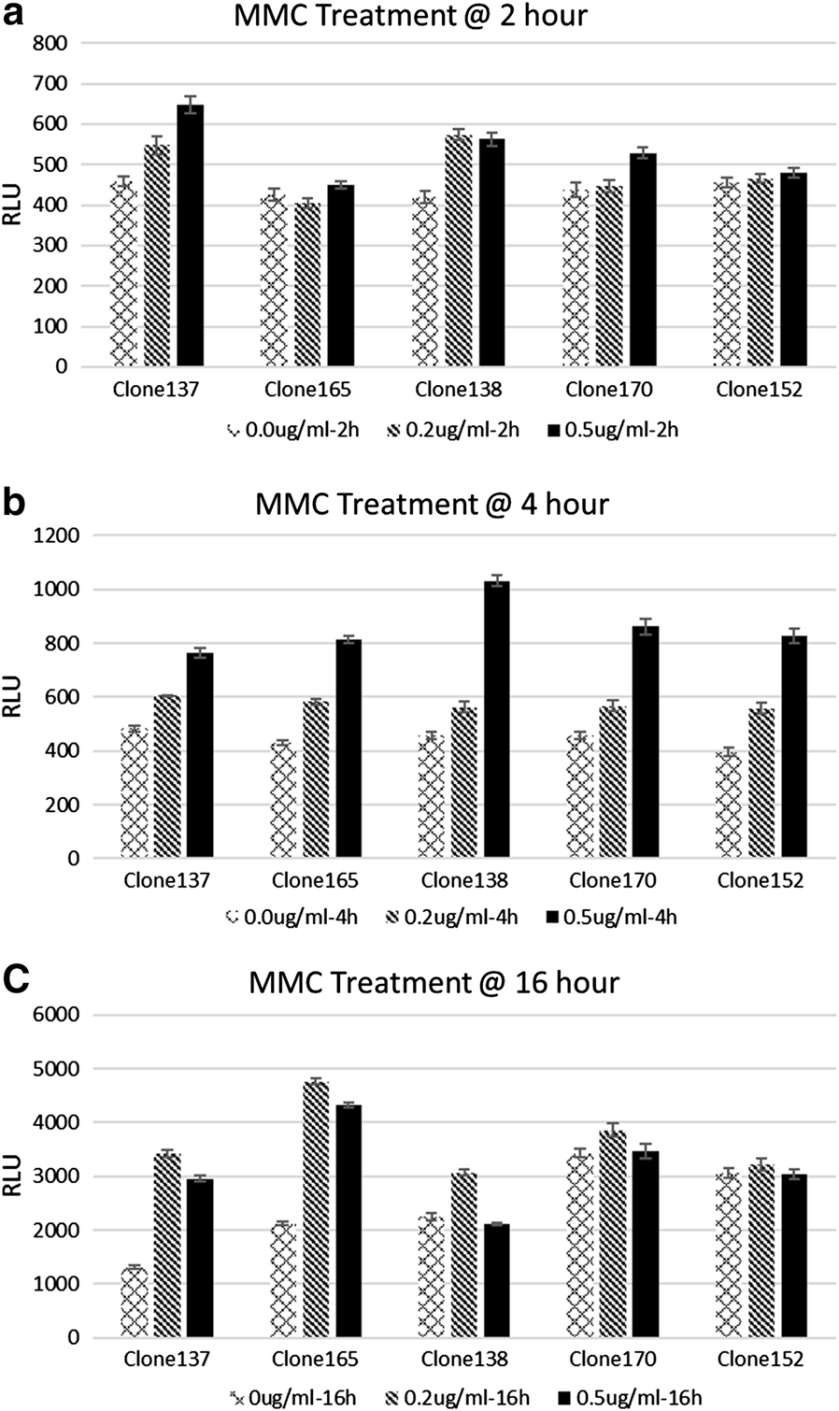
Analysis of LexA-containing clones in response to mitomycin C treatment. From protein-bound genomic DNA fragment libraries, five clones containing LexA binding sequences were identified through sequencing and TFBS motif search. These clones were selected and treated with 0, 0.2 and 0.5 μM mitomycin C for 2 (**a**), 4 (**b**), and 16 h (**c**) respectively. Cells were collected for luciferase analysis

In order to conduct functionality of the predicted TFBS in reporter vector, we first chose *lexA* as our testing target since the LexA DNA binding site was recognized to appear more frequently than others, and was predicted to be located on several gene promoter sequences, including *kdo*, *fimZ*, *dtpD*, and *ElbB*. Furthermore, LexA is widely studied and is well known to be induced by environmental stress (Maslowska et al. 2019; Kreuzer 2013). Five clones containing LexA binding sites were selected for functional tests of MMC-mediated activation of LexA: clone 137 *dtpD*, clone 138 *elbB*, clone 152 (non-coding Pseudo gene), clone 170 *kdo* and clone 165 *fimZ*, which was previously reported to be regulated by LexA (Saini et al. 2009). These clone plasmids were transformed into DH5a, and inoculated and treated with 0, 0.2 and 0.5 μM MMC for 2, 4 and 16 h respectively (Fig. 2), and cell lysates were prepared for luciferase analysis. 2 h treatment did not show significant induction rate (Fig. 2a). Even though all of these clones showed the clear induction at 0.5 μg/mL MMC for 4 h treatment (Fig. 2b), the induction patterns showed a slight difference in terms of the condition for highest induction: clone 137 and clone 165 showed highest induction at 0.2 μg/mL for a 16 h treatment (Fig. 2c), while clone 138, clone 152 and clone 170 showed highest induction at 0.5 μg/mL for 4 h treatment (Fig. 2b). The results of the clones containing LexA binding sequences were confirmed to be induced by MMC with luciferase assays, since the LexA binding sequences in these clones come from different gene promoter regions, which may affect the responding pattern of MMC treatment.

**Fig. 3:**
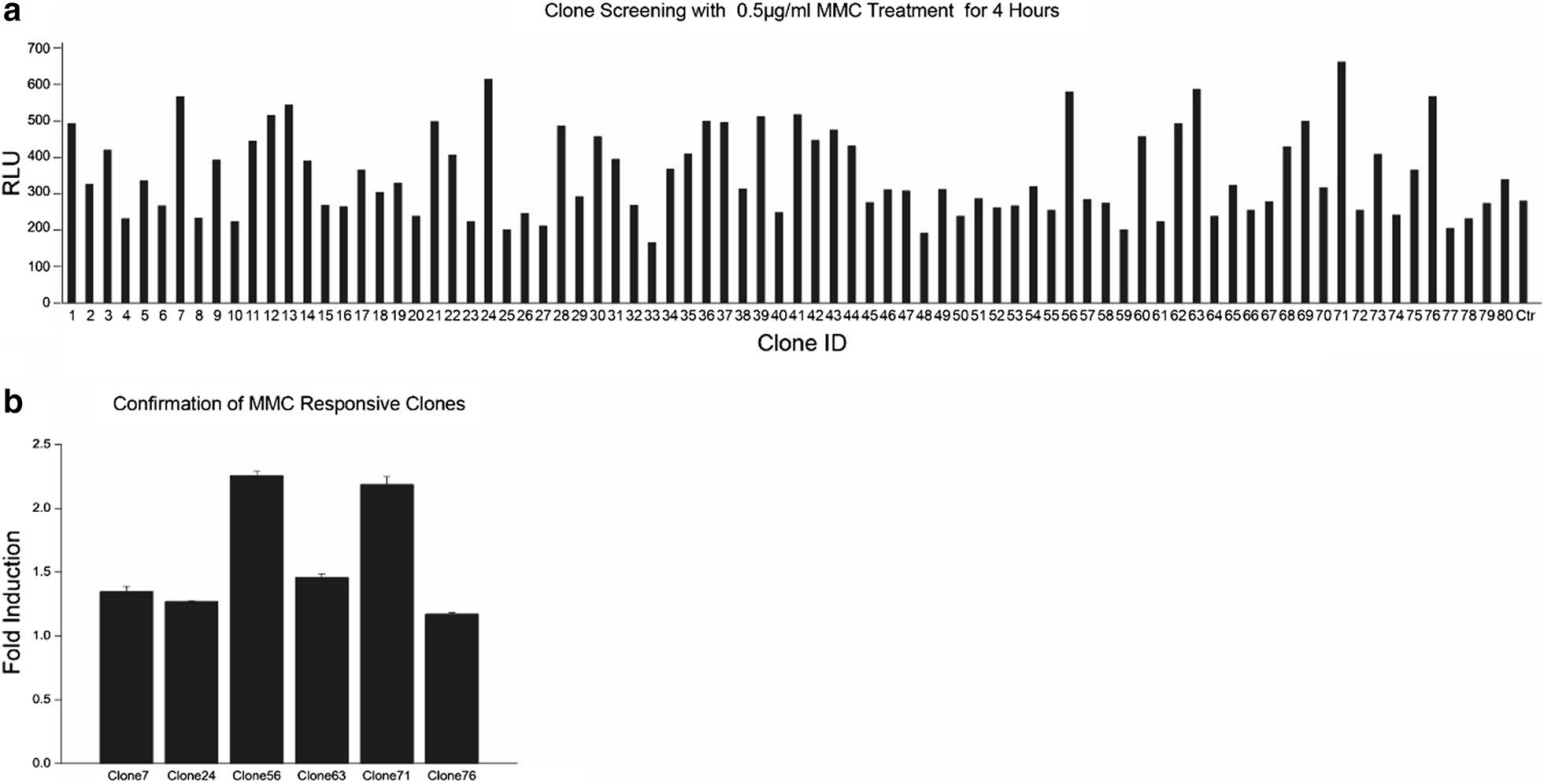
Directly functional screening of mitomycin C-responsive clones. Eighty clones randomly selected from generated library, cultured them with 0.5 μM mitomycin C treatment for 4 h, and subject to luciferase analysis (**a**). Six clones with luciferase activities > 550 RLU were selected for induction assay with and without mitomycin C treatment (**b**)

To further demonstrate the feasibility of direct library screening without prior information, 80 clones were randomly selected from libraries. We chose treatment conditions of 0.5 μg/mL for 4 h since under these conditions all of the LexA binding site containing clones showed a significant induction. Cell lysates were prepared and subjected to luciferase analysis. As shown in Fig. 3a, 80 clones were first screened with MMC treatment, and 6 clones with higher luciferase activities (> 550 RLU (relative light unit)) were selected for induction assay. Two clones, clone 56 and clone 71, were identified with twofold LexA induction (Fig. 3b). Sequencing analysis with BLAST search (NCBI, USA) revealed that clone 56 is an unknown target, and clone 71 contains *elbB*. Both clones were further analyzed with BPROM and predicted with a LexA binding site. The predicted LexA binding sequence in clone 71 is TTTTTTTA; while clone 138 is TAAATTATTAT.

**Fig. 4:**
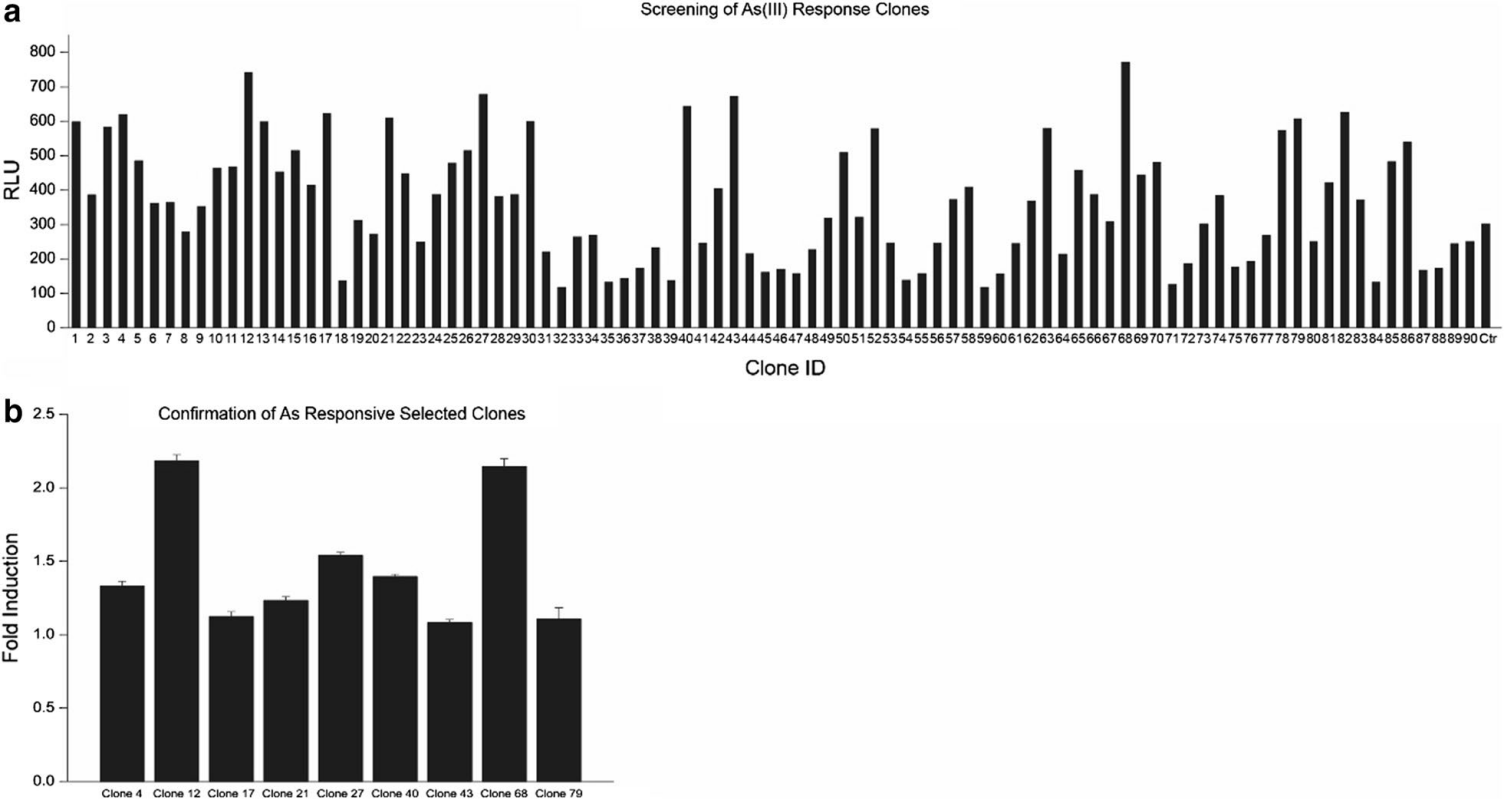
Directly functional screening of arsenite-responsive clones. Ninety clones from library were randomly selected, cultured with 5 μM arsenite treatment for 2 h, and subject to luciferase analysis (**a**). Nine clones with luciferase activities > 600 RLU were then selected for arsenite induction assay (**b**)

**Fig. 5:**
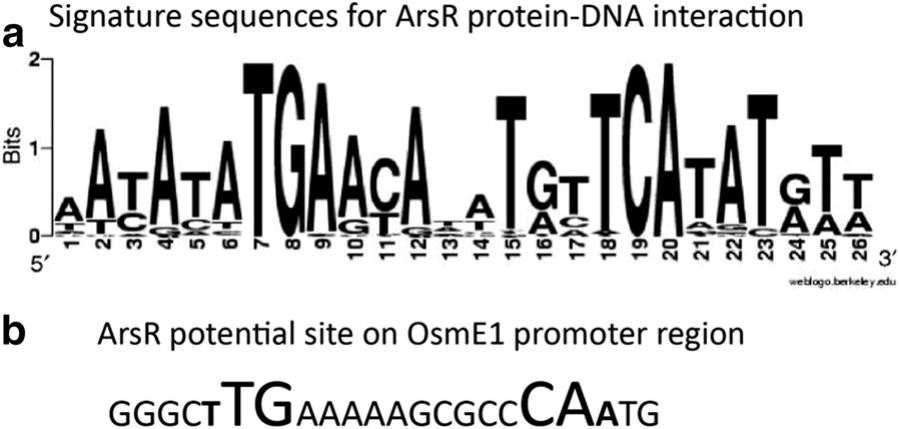
Signature sequences for *arsR* protein–DNA interaction and OsmE1 promoter region

To validate our direct screening function of libraries, we utilized *arsR* as another screening target, which we have widely studied in our recent publication (Chen et al. 2019). Another 90 clones from libraries were cultured and treated with 5 μM arsenite for 2 h based our previous optimal conditions. Nine clones showing high luciferase activities (> 600 RLU) (Fig. 4a) were then selected and analyzed thoroughly with individual arsenite induction assay. Two clones, clone 12 and 68, were confirmed to have greater than twofolds induction. The plasmids were prepared from clone 12 and 68 and then subjected to sequencing. Through NCBI BLAST search, clone 12 revealed *osmE1* and clone 68 revealed *arsR.* Both clones were unable to be analyzed with the promoter prediction program BPROM as the program does not contain ArsR binding sequences, although Arsenite-mediated induction of ArsR is well-documented (Chen et al. 2017, 2019; Bose et al. 2006; Kostal et al. 2004). The *arsR* binding site on ArsR found in this study TTAAATCATATGCGTTTTTGGTT was the identical to the published one (Xu et al. 1996). The potential ArsR binding site on *osmE1* were predicted to be GCt**TG**AAAAAGCGCC**CAa**TG based on reported consensus sequence, tTGxxxx xx xxxxCAa (Busenlehner et al. 2003) shown in Fig. 5.

**Fig. 6:**
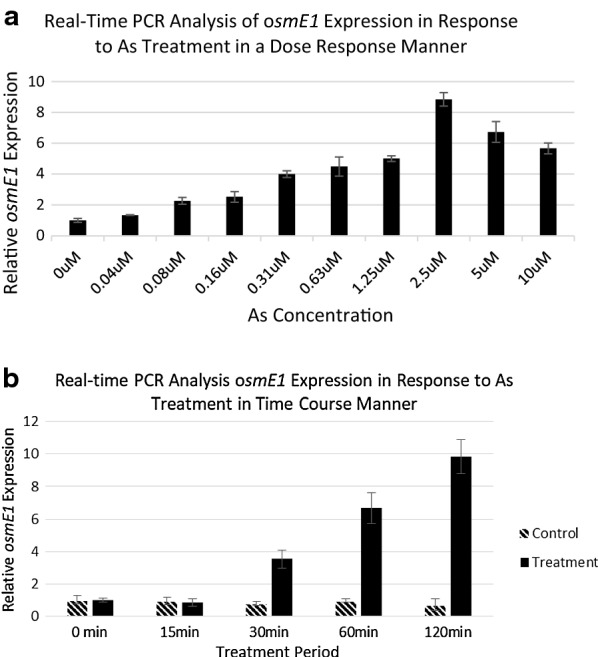
Quantitative analysis of *osmE1* gene expression with RT-PCR. DH5α were treated with 0, 0.04, 0.08, 0.16, 0.31, 0.63, 1.25, 2.5, 5, and 10 μM arsenite for 2 h (**a**) or with 2.5 μM arsensite for 0, 15, 30, 60, and 120 min (**b**). RNA was isolated from DH5α and reverse transcribed to cDNA with AMV. Real-time PCR with SYBR green was performed with ABI PRISM 7000 Sequence Detection System. Quantification of *osmE1* RNA was normalized using reference 16S rRNA, *gry*A and mGOD

Since *osmE1* is not well studied and is newly discovered in our study, this gene induction by arsenite treatment needs to be further investigated. To analyze arsenite-mediated induction of *osmE1* gene expression, we utilized real time RT-PCR quantitative measurement in time and dose course. For dose response assays, DH5α cells were treated with 0, 0.04, 0.08. 0.16, 0.31, 0.63, 1.25, 2.5, 5, and 10 μM arsenite for 2 h. Total RNAs were prepared, and reverse transcribed to cDNA. SYBR Green PCR reactions were performed in duplicate, and the mean Ct values for each trial were calculated. As shown in Fig. 6a, the treatment with 2.5 μM of arsenite yielded the highest induction of *osmE1* gene expression. Next, we examined the time-course response of *OsmE1* gene expression to 2.5 μM arsenite for periods of 0, 15, 30, 60 and 120 min. The samples were collected at the indicated time points and quantification of *osmE1* gene expression normalized using the references. The results revealed that the 120 min treatment yielded the highest induction, ninefolds, of *osmE1* gene expression (Fig. 6b).

## Discussion

Bacteria biosensors act as a new class of detectors to produce a detectable signal upon activation of a promoter reporter gene induced by specific stimuli, which have been used for monitoring environmental pollutants such as heavy metals or pesticides (Gutiérrez et al. 2015). The key component of whole-cell biosensors is the reporter (Gui et al. 2017), consisting of a promoter/operator and a reporter gene. Therefore, it is crucial to find a responding promoter/operator in a high throughput method from surviving microbes in an environment containing a target pollutant. The current bacteria reporter biosensors are only for the known toxin substance-induced TFBS constructed reporter system, and cannot be used for discovering a TF and the associated TFBS for a novel substance.

This study presents a novel approach to enriching protein-bound genomic DNA fragments for the construction of luciferase libraries conducting directly functional screening to identify substance-responsive TFBS elements. This dramatically reduces time and labor in the screening of unknown TFBS elements in response to a potential toxin substance. It has been widely known that there are around 300 TFs and seven sigma factors in the *E. coli* genome (Pérez-Rueda and Collado-Vides 2000; Tripathi et al. 2014). Our protein bound enriched DNA libraries displayed 131 TFBS containing clones from screening 280 clones based on sequencing analysis and bacteria TFBS prediction software BPROM, and identified two well-studied ArsR (Chen et al. 2017, 2019; Bose et al. 2006; Kostal et al. 2004) and FimZ (Saini et al. 2009) among these TFs, which demonstrating our libraries are highly enriched with useful TFBS information. In addition, through luciferase assay, the same TF (such as LexA) on the promoter region with different binding sequences were shown to have various induction patterns, therefore the libraries can not only obtain a specific TF binding motif, but also provide multiple promoter associated binding sequences with different induction patterns, which may offer possibilities to develop more sensitive and selective stress substance screening system. Through direct functional screening, we were able to obtain MMC-responsive *lexA* clones and As-responsive *arsR* and *osmE1* clones. These results showed that our functional libraries can be utilized to efficiently screen and discover the responsive clones under stress substance stimulation. Our library screening does not require the prior knowledge of the target microbial genome or any known transcription factor, therefore our libraries have great potential to be used for identifying a specific TF binding site of a given substance, and developing functional screening methods for unknown microbes with very limited physiological and genomic information.

Studies demonstrate that arsenite can mediate ArsR induction, which is well-documented in literature (Chen et al. 2017, 2019; Bose et al. 2006; Kostal et al. 2004). ArsR, belonging to the Smt/ArsR family, is a regulatory protein that controls the expression of the genes involved in arsenical resistance via interaction with the arsenic-responsive operon (Chen et al. 2017). Due to the abundant presence of ArsR binding sequences in microbial chromosomes, the alignment of these binding sequences via comparison and analysis leads to the identification of a binding consensus sequence (Saini et al. 2009). SmtB/ArsR binding sequences share a conserved 12-2-12 palindrome (Kostal et al. 2004). Our recent study indicated that among the inverted repeat, TC and GA are critical to ArsR binding (Chen et al. 2019). Interestingly, we found that OsmE1 is also a target capable of regulation by arsenite, although this has been shown in only one previous study (Patel 2005). This study reported that the Identification of the arsenic binding-protein fractions with arsenic analysis revealed two low molecular weight proteins, which one of them being OsmE1. Cells under arsenate stress conditions could allow the expression of *osmE1*. Further studies need to determine how many genes are induced under arsenic stress, how they are regulated by arsenite, and what function they play in response to arsenic stress.

Our *E. coli* protein-bound DNA enriched functional library technology can easily be adapted to mammalian TFBS identification; however, mammalian transcriptional regulation is much more complicated than bacteria transcriptional regulation as there are more than 2000 TFs for mammals (Brivanlou and Darnell 2002). Luciferase-based screening may be time-consuming to assay individual clones. GFP reporter can replace luciferase reporter to construct libraries, so that the differentially expressed reporter genes can be easily identified through fluorescence-activated cell sorting (FACS) to sort the interesting population in response to a certain treatment. Our protein-bound enriched functional library technology has a wide application for TFBS identification of unknown transcriptional regulation in prokaryotic and eukaryotic system.

## Data Availability

All data and materials are available.
